# The Vital Role Played by Deferiprone in the Transition of Thalassaemia from a Fatal to a Chronic Disease and Challenges in Its Repurposing for Use in Non-Iron-Loaded Diseases

**DOI:** 10.3390/ph16071016

**Published:** 2023-07-18

**Authors:** George J. Kontoghiorghes

**Affiliations:** Postgraduate Research Institute of Science, Technology, Environment and Medicine, Limassol 3021, Cyprus; kontoghiorghes.g.j@pri.ac.cy; Tel./Fax: +357-26-272076

**Keywords:** deferiprone, thalassaemia, iron overload, chelation therapy, drug repurposing, antioxidants, metal detoxification, neurodegenerative diseases, orphan drugs

## Abstract

The iron chelating orphan drug deferiprone (L1), discovered over 40 years ago, has been used daily by patients across the world at high doses (75–100 mg/kg) for more than 30 years with no serious toxicity. The level of safety and the simple, inexpensive synthesis are some of the many unique properties of L1, which played a major role in the contribution of the drug in the transition of thalassaemia from a fatal to a chronic disease. Other unique and valuable clinical properties of L1 in relation to pharmacology and metabolism include: oral effectiveness, which improved compliance compared to the prototype therapy with subcutaneous deferoxamine; highly effective iron removal from all iron-loaded organs, particularly the heart, which is the major target organ of iron toxicity and the cause of mortality in thalassaemic patients; an ability to achieve negative iron balance, completely remove all excess iron, and maintain normal iron stores in thalassaemic patients; rapid absorption from the stomach and rapid clearance from the body, allowing a greater frequency of repeated administration and overall increased efficacy of iron excretion, which is dependent on the dose used and also the concentration achieved at the site of drug action; and its ability to cross the blood–brain barrier and treat malignant, neurological, and microbial diseases affecting the brain. Some differential pharmacological activity by L1 among patients has been generally shown in relation to the absorption, distribution, metabolism, elimination, and toxicity (ADMET) of the drug. Unique properties exhibited by L1 in comparison to other drugs include specific protein interactions and antioxidant effects, such as iron removal from transferrin and lactoferrin; inhibition of iron and copper catalytic production of free radicals, ferroptosis, and cuproptosis; and inhibition of iron-containing proteins associated with different pathological conditions. The unique properties of L1 have attracted the interest of many investigators for drug repurposing and use in many pathological conditions, including cancer, neurodegenerative conditions, microbial conditions, renal conditions, free radical pathology, metal intoxication in relation to Fe, Cu, Al, Zn, Ga, In, U, and Pu, and other diseases. Similarly, the properties of L1 increase the prospects of its wider use in optimizing therapeutic efforts in many other fields of medicine, including synergies with other drugs.

## 1. Introduction

The development of new pharmaceuticals is usually undertaken by large, multinational companies, and the process requires many years and great expenditure to fulfil the necessary drug regulatory requirements. In contrast, the repurposing of already regulatory approved drugs requires less effort, but it is also a very challenging task, especially in research investigations for the development of therapies for serious and life-threatening diseases and also for emergency medicines [1,2,3,4,5]. This challenge is particularly important and pressing for many categories of patients where effective therapies have not yet become available. These categories include millions of patients with cancer and neurodegenerative, infectious, and other diseases [1,2,3,4,5,6,7,8,9]. Similarly, drug repurposing is very important for many categories of patients with orphan diseases, particularly the millions of patients in developing countries, where health resources are scarce [10,11,12].

Some of the major categories of diseases affecting humans are those related to the abnormal metabolism of iron, an essential metal required by all cells of the body and also by microbes and cancer cells. Iron is also required for many biological processes involving a large number of iron-containing enzymes participating in a variety of metabolic pathways and also for essential physiological processes including respiration, energy transduction, haemopoiesis, growth, and development [13,14,15,16].

Billions of people are known to be suffering from diseases of iron metabolic imbalance, including iron deficiency anaemia and iron overload [17,18,19,20,21,22,23,24,25]. In contrast to its vital role, iron has also been implicated in many diseases of tissue damage involving redox active labile iron. In this case, the basic mechanism of toxicity is related to the role or association of iron as the main biological catalyst in free radical pathology [26,27,28,29,30].

The iron chelating drugs deferiprone (L1), deferasirox (DFRA), and deferoxamine (DF), which are widely used in the treatment of transfusional iron loading conditions, are included in the orphan drug category and have the prospect of being repurposed for the treatment of many other clinical conditions [12,31,32,33,34,35]. Both L1 and DFRA are orally active, whereas DF has to be administered subcutaneously or intravenously to be effective [31,32,33,34,35]. Many investigations, including clinical trials, have been previously carried out using chelating drugs for their possible use in clinical conditions other than transfusional iron overload. However, in many such cases, the results were disappointing, mainly because of toxicity or the use of unsuitable methodologies and doses [36,37,38].

General limitations of drug repurposing include pharmacological and toxicological investigations, which may be related to the proposed drug’s properties or the disease characteristics. Some of these aspects include, for example, drug posology and protocols, toxicity concerns, compliance with the method of administration, special factors and limitations related to the underlying disease, and the overall risk/benefit assessment in comparison to existing therapies.

One of the major concerns regarding chelating drug repurposing is related to the toxicity limitations identified in the cases of DFRA and DF in different categories of patients with normal iron stores, where it was recommended by the manufacturers that these drugs should not be administered to patients with serum ferritin lower than 0.5 mg/L [38,39,40]. In contrast, in the case of L1, no such restrictions are imposed for its use, and the drug can be available for investigations related to the treatment of a wider range of diseases [41]. There are many other advantages to the use of L1 in comparison to DF and DFRA, but also some toxicity concerns, which have to be considered and monitored during clinical and other investigations.

**Figure 1:**
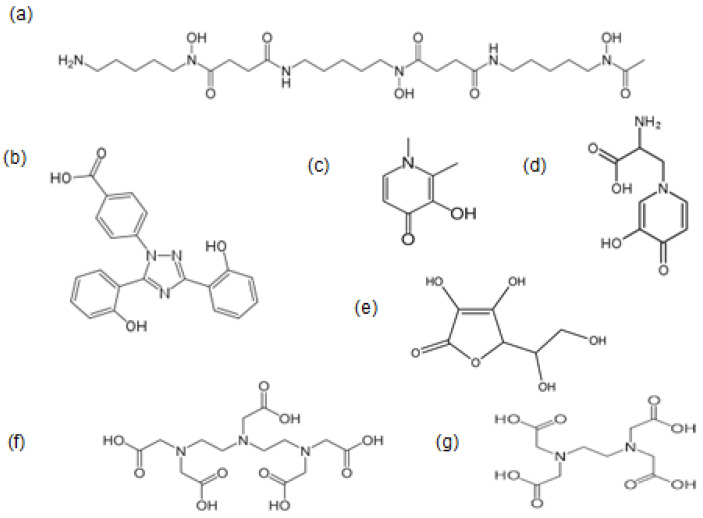
The chemical structure of iron and other metal chelating drugs. Deferoxamine (**a**), deferasirox (**b**), and deferiprone (**c**) are widely used for the treatment of iron overload in thalassaemia and other conditions. Mimosine (**d**) and ascorbate (**e**) are natural phytochelators consumed by humans. Diethylenetriaminepentacetic acid or DTPA (**f**) is used for plutonium decontamination, and ethylenediaminetetracetic acid or EDTA (**g**) is used for general metal detoxification and in alternative medicine.

The chemical, pharmacological, and other properties of drugs are crucial for identifying their mode of action, toxicity, and therapeutic characteristics in each disease [34,35]. In this context, the unique properties of L1 and their implications for therapeutic activity in different diseases have been investigated and, where appropriate, compared to other chelating drugs (Figure 1). Similarly, the effect of drug interactions and drug combinations is discussed within the framework of optimizing therapeutic activity and minimising toxicity implications in different clinical conditions. Furthermore, other factors appear to affect the efficacy and toxicity of L1, such as the selection of drug posology in different clinical conditions based on diagnostic criteria and other aspects of the underlying disease.

The prime objective of this review is to identify the unique properties and other characteristics of L1, which helped in the transition of thalassaemia from a fatal to a chronic disease, and to apply (or, where possible, to improve) these for repurposing in other diseases. The prospect of wider use of L1 in different diseases, taking into consideration the limitations of the mode of activity, low cost, and other drug characteristics, could benefit many patients, including different orphan disease patient categories who do not receive effective drug treatments at present [12].

## 2. The Unique Clinical Characteristics of Deferiprone

**Figure 2:**
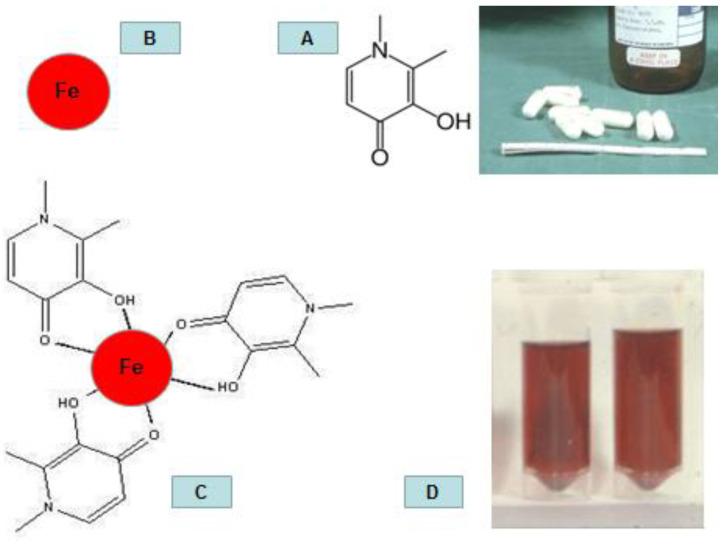
The pharmacological activity of deferiprone in iron-loaded patients. The first pharmaceutical preparation of deferiprone (L1) in gelatine capsules was used in clinical trials in London, UK. Each gelatine capsule contained 500 mg of white crystalline solid of L1 (**A**). Deferiprone can bind and remove iron (Fe) from different iron pools and organs (**B**) and form a tris L1 iron complex, which has an orange/red colour (**C**). The orange/red-coloured tris L1 iron complex is excreted in the urine, as shown in the red urine samples of iron-loaded thalassaemia patients treated with L1 (**D**).

Deferiprone, discovered about forty years ago (1981), was the first oral iron chelating drug to be introduced for the treatment of iron overload in thalassaemia and other transfusional iron loading conditions worldwide, following approval by the regulatory authorities for clinical use (first in India in 1995, then in the EU in 1999, and then in the USA in 2011) [42,43,44,45,46]. Even before approval by the regulatory authorities, thousands of thalassaemia patients were treated with L1 worldwide in clinical trials and on compassionate use permissions a few years following the results of the first clinical trials reported in 1987 in London, UK (Figure 2) [47,48,49,50,51,52,53,54,55,56,57,58,59]. The main reasons for the early availability of L1 to many thousands of thalassaemia patients worldwide prior to regulatory approval were the low compliance and toxicity effects experienced by many patients using the only other available chelating drug at that time, DF, which was administered subcutaneously 8–10 h per day [47,48,49,50,51,52,53,54,55,56,57,58,59]. Equally important was the wide access to L1 due to a new, simple, inexpensive synthesis, as well as long-term stability and storage at room temperature [60].

In addition to the inexpensive chemical synthesis and high compliance, several other factors contributed to the wider availability of L1 in comparison to DF before registration, including its high efficacy in iron removal and its low toxicity at the identified efficient selected doses [48,49]. Regarding the latter, L1 is considered one of the safest chronically administered drugs on a daily basis at one of the highest doses per body weight (75–100 mg/kg) used in medicine [46]. For example, patients with a body weight of 75 kg receiving the highest approved dose (100 mg/kg) are treated, overall, with 7.5 g of L1 daily divided into two or three doses.

In more than 30 years of clinical use and monitoring of L1 in different categories of patients, all the reported toxic side effects of L1 are considered manageable and reversible. The most serious toxic side effects of L1 include agranulocytosis and neutropenia, affecting less than 1% and 5% of patients, respectively. Less serious toxic side effects include gastric intolerance, musculoskeletal and joint pains, and zinc deficiency [35,61,62,63,64,65]. Toxicity vigilance and prophylactic measures were introduced at the early stages of the clinical use of L1. In this context, and in addition to regular clinical and biochemical monitoring, a weekly or fortnightly blood count is recommended for prophylaxis against agranulocytosis and neutropenia [65]. Similarly, the use of zinc supplements is also recommended for prophylaxis in patients on long-term treatment with L1 [65,66].

The efficacy of L1 in removing sufficient amounts of excess iron for achieving a negative iron balance in transfusional iron-loaded patients has been recognised since the initial clinical trials, where stepwise increments of doses from 10 mg/kg/day to a maximum of 110 mg/kg/day were used [49,50]. The doses selected in patients were based on previous animal studies, where repeated administrations of L1 have shown further iron excretion increases without reaching a plateau or saturation point [42,67,68,69,70,71,72,73]. Similar results from the repeated administration of L1 were reported in iron-loaded patients, where, in general, iron excretion depended on the dose and the iron load of patients. For example, in intensive iron chelation studies in heavily iron-loaded thalassaemia patient using repeated administration of L1 to a maximum of 250 mg/kg/day, a record maximum total of 325 mg/day of iron was excreted in the urine (Figure 2) [49,50,74].

Most thalassaemia patients are born in developing countries where health resources are scarce, and the majority of patients receive insufficient or no effective treatment. The life expectancy of thalassaemia patients without any treatment is limited to about 2–3 years as a result of ineffective erythropoiesis and severe anaemia [19,20,21,22]. Treatment of the anaemia with the introduction of chronic red blood cell transfusions can increase life expectancy to about 20 years; the main cause of death is congestive cardiac failure as a result of excess iron deposition in the heart from the multiple transfusions [22,75]. Life expectancy has increased with the introduction of iron chelation therapy using subcutaneous DF, where, again, the main cause of death is congestive cardiac failure [75,76,77]. A monitoring report of thalassaemia patients treated with subcutaneous DF prior to the introduction of L1 in the UK estimated the mean survival of patients to be 35 years [78].

Following the introduction of L1 and the L1/DF combinations, increased survival and a reduction in mortality were reported in thalassaemia patients treated in Cyprus and other countries [79,80,81]. Many thalassaemia patients treated with L1 and the L1/DF combination have since then surpassed 50 years of age, with a life expectancy reaching that of the general population; patients have also gone on to have children and grandchildren [65,82]. The transition of thalassaemia from a fatal to a chronic disease has been achieved mostly as a result of the ability of L1 to remove excess iron from the heart efficiently and to reduce cardiac failure, which was the main cause of mortality prior to its introduction [83,84,85,86,87,88,89,90]. This unique property of L1 in comparison to the other chelating drugs was identified following the introduction of new magnetic resonance imaging (MRI) techniques (MRI T2 and T2*), which can be used to identify the level of excess iron in the heart and also the other organs [91,92,93,94,95,96].

Another major finding in relation to the diagnostic use of MRI T2 and T2* and other iron load diagnostics is the monitoring of the rate of iron removal effects of chelating drugs. In particular, by utilising these diagnostic techniques, it was possible to identify iron chelation protocols, such as the International Committee on Chelation (ICOC) protocol, which can efficiently remove all excess iron in chronically transfused thalassaemia patients [85,97,98,99,100]. This is another unique property of L1, which is based on its high efficacy of iron removal, which can result in the reduction of iron stores and the maintenance of normal MRI T2* signal intensity levels in the heart, liver, and spleen, and also normal serum ferritin levels in thalassaemia patients [97,98,99,100,101]. Similar effects have been shown using the ICOC and other protocols of the L1/DF combination. In particular, the L1/DF combination protocol has recently been shown to be more effective than L1 and DFRA monotherapy in clearing excess pancreatic iron in thalassaemia patients [102].

The ICOC protocols are based on personalised chelation therapy options of either L1 (75–100 mg/kg/day) monotherapy or a combination therapy of L1 (75–100 mg/kg/day) and DF (40–60 mg/kg at least three times per week) [85,97,103,104]. However, there is no overall consensus among physicians on the selection of chelation protocols in different countries and clinics where various chelation options are available, which include different L1, DF, and DFRA monotherapies and their combinations. Unsurprisingly, the majority of such protocols are not sufficiently effective, which, in most cases, leads to patients receiving suboptimal chelating drug doses and maintaining increased iron load levels. However, some patients experiencing toxicity or low levels of efficacy in iron removal with one of the chelating drugs have an alternative option and can benefit from the use of any of the other chelating drugs or their combination [105,106,107].

## 3. The Pharmacological and Metabolic Properties of Deferiprone

The efficacy and toxicity of L1 and also of other drugs are mostly defined by their pharmacological and metabolic properties, which are also influenced by their molecular and structural characteristics. In the case of L1, the small size, heteroaromatic structure, and hydrophilic properties appear to facilitate its rapid gastrointestinal absorption following oral administration [108,109,110]. Furthermore, the absorption and blood appearance within minutes ensures that L1 can rapidly reach almost all major organs and exert its iron chelating and other pharmacological activities. In this context, L1 can rapidly chelate mononuclear labile forms of toxic iron species, such as non-transferrin-bound iron and intracellular low-molecular-weight iron, as well as other protein-associated forms of iron, from iron-loaded patients (Figure 2) [111]. Deferiprone is also found in the saliva of treated individuals and is likely to be found in other secretions, including the milk of lactating female patients [112,113]. The rapid absorption of L1 can be affected by interactions with food components, metal ions, and drugs, such as aluminium-containing antacids [114,115].

Following iron mobilisation, a characteristic orange/red-coloured urine is excreted in the case of iron-loaded patients receiving L1, which has the same colour as the L1 iron complex (Figure 2). In contrast, in non-iron-loaded individuals with normal iron stores receiving equivalent single doses of L1 (e.g., 30 mg/kg), only a few mg of iron is excreted, which does not give the characteristic deep orange/red-coloured urine observed in iron-loaded patients [74,116]. Furthermore, there have been no reports of increased faecal iron excretion during iron balance studies, and L1 and its metabolite have not been detected in faecal samples of thalassaemia patients treated with L1 [74,116].

**Figure 3:**
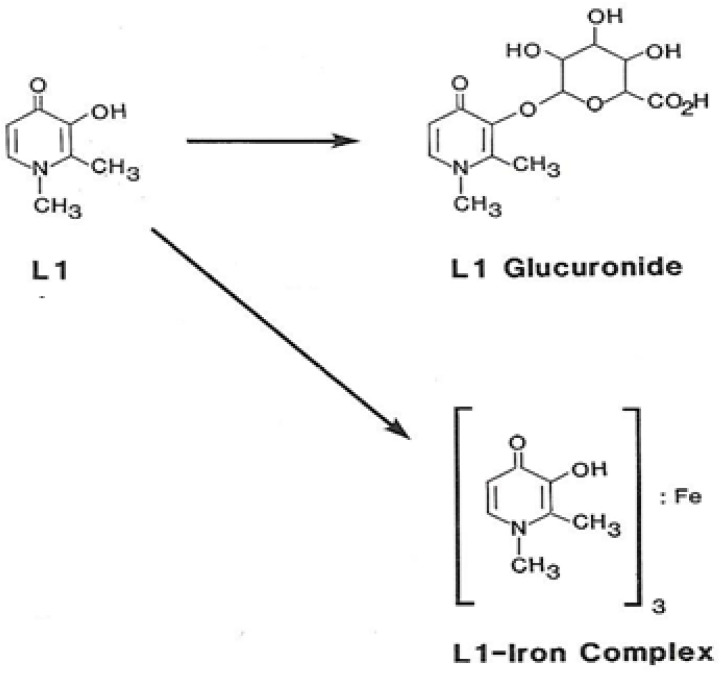
The major metabolic pathways of deferiprone in humans. Deferiprone is mostly metabolised to the deferiprone (L1) glucuronide conjugate, which is the major metabolite in individuals with normal iron store levels. The covalent binding of glucuronic acid at the 3-OH iron binding site of L1 prevents the formation of the L1 iron and other metal complexes. The tris L1 iron complex is another major metabolic product of L1, which is mostly formed in iron-loaded patients. Minor L1 metal metabolites may also be formed with other metals, such as zinc and aluminium.

Most of the administered L1 in patients and normal volunteers is metabolised to a glucuronide conjugate, which has no chelation properties and is also excreted in the urine. It should be noted that the 3-hydroxyl group of L1 is involved both in iron chelation and also during glucuronic acid conjugation. Overall, it appears that iron binding by L1 prevents glucuronidation and vice versa. In this context, the rate of iron chelation is faster than glucuronidation, and the presence of more chelatable iron forms in an individual reduces the rate of glucuronidation of L1 [74,116,117] (Figure 2 and Figure 3).

The same effects on glucuronidation are expected in the chelation of other metals by L1, such as aluminium [115,117,118,119]. The rate of glucuronidation is also expected to be affected by other pharmacological and metabolic factors, including the absorption, distribution, metabolism, elimination, and toxicity (ADMET) of L1, which is also observed in all other drugs and is different in each patient. In this context, a variable rate of glucuronidation has been observed in a monitoring metabolic study of iron-loaded patients treated with L1, and, in some cases, glucuronidation was absent [117]. Similarly, pharmacological and other factors, such as drugs affecting enzymes related to glucuronidation, drugs including probenecid, and other drug interactions, can influence this metabolic process and the rate of glucuronidation of L1 [120,121,122].

Pharmacokinetic studies indicate that following oral administration, the clearance of L1 from blood circulation can take about 6 h, and clearance of its glucuronide conjugate takes about 9 h [108,109,110,116]. The pharmacokinetic profile of L1 and its glucuronide conjugate allows the repeated administration of the drug several times a day, which, in clinical practice, usually allows the minimum administration of L1 twice or three times a day. However, some studies have shown that increasing the number of administrations and using higher overall doses of L1 could further increase iron excretion and potentially be used in intensive chelation protocols [49,74]. In contrast, DF and DFRA can only be administered once daily due to restrictions imposed by the pharmacokinetic and pharmacodynamic properties of the drugs [122,123,124,125].

Pharmacological and metabolic properties are important parameters influencing the efficacy of drugs including L1 (Table 1). Similar influences on efficacy are also exerted by the toxicity and posology of the administered drug. For example, regarding posology, it has been shown in general that the higher the dose used and the higher the iron load of a patient, the higher the amount of iron that is mobilised and excreted [49,74,122]. Similarly, different modes of pharmacological action and interactions with iron-containing proteins affecting associated metabolic pathways in health and disease are observed in vivo by L1 and are dependent on the concentration of the drug [111]. However, the general therapeutic activity is overall more complex because metallomic, genomic, proteomic, metabolomic, pharmacogenomic, and other factors can also influence the therapeutic outcome in the case of L1 and also of other drugs [126,127,128,129].

## 4. The Unique Effects on Proteins by Deferiprone and Therapeutic Implications

There are many interactions of L1 with iron-containing and other proteins and different associated effects, all of which appear to influence its therapeutic activity and toxicity. Some of these effects have been discovered in the early screening studies for the identification of L1′s mode of action and also in comparison to DF and other chelators (Table 1) [42,111].

One of the most important and unique properties of L1 not found in any other drugs is the ability to remove iron from the iron transport protein transferrin found in blood, which delivers iron to all the cells of the body, including microbial and cancer cells [130,131,132,133]. The removal of iron from diferric transferrin is crucial in iron overloading conditions because it can result in an increase in iron excretion and also a decrease in the rate of excess iron delivery by transferrin to the cells, including a decrease in the rate of iron deposition and subsequent damage to sensitive organs, such as the heart [134,135,136]. Similarly, the reduction of iron delivery by transferrin and the inhibition of associated metabolic processes, including the functioning of transferrin receptors, are considered important targets for the design and development of new potential pharmaceuticals against microbial infections and cancer [136,137,138].

Another unique property associated with L1 but not with any other drugs is its ability to remove iron from lactoferrin, the sister protein of transferrin, which is found in secretory fluids, such as milk, saliva, tears, and nasal and vaginal secretions. Lactoferrin is also a component of the immune system found in the secondary granules of neutrophils. It has antimicrobial properties and plays a major role in infectious and inflammatory diseases [139,140,141,142,143]. The interactions of L1 with lactoferrin are likely to facilitate its physiological functions, including antimicrobial and anti-inflammatory activities, which cannot be fulfilled if lactoferrin is saturated with iron [143].

**Figure 4:**
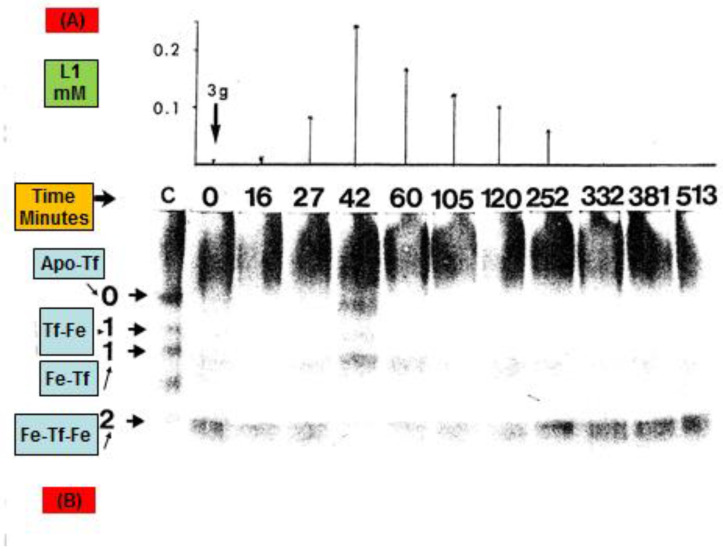
Iron removal from diferric transferrin following the administration of deferiprone in a thalassaemia patient. A 21-year-old male thalassemia patient with serum ferritin of 5.9 mg/L was treated with 3 g of deferiprone (L1). A total of eleven blood samples were obtained from the patient over a period of 513 min following the administration of L1 at time 0. In each sample, the concentration of L1 was estimated using high performance liquid chromatography (HPLC), as shown in (**A**). Similarly, for each sample, the transferrin (Tf) iron saturation was monitored using urea polyacrylamide gel electrophoresis (UPAGE), as shown in (**B**). The fourth serum sample at 42 min shows the maximum L1 concentration (0.25 mM) and maximum iron mobilisation from the diferric Tf (Fe-Tf-Fe), with gel bands corresponding to apoferric transferrin (apoTf) and monoferric C-terminal (Tf-Fe) and N-terminal (Fe-Tf) Tf. After this period, L1 is steadily cleared from the blood, and the iron saturation of Tf is restored. Adapted with permission from [134].

In vitro and clinical studies in thalassaemia patients treated with L1 have shown that iron removal from diferric transferrin is L1-concentration-dependent and can only be accomplished if the L1 concentration is higher than about 0.15 mM in blood (Figure 4) [144,145]. Conversely, L1 can be used as an iron donor for apo-transferrin. In this context, apo-transferrin can compete and take iron from the L1 iron complex both in vitro and also in vivo; for example, in normal individuals treated with L1 [134,136,146]. In particular, this is the mechanism of action proposed for L1 in the treatment of the anaemia of chronic disease, such as rheumatoid arthritis, where L1 has been shown to remove iron from macrophages of the reticuloendothelial system and donate it to transferrin, which then can transfer it to the haemopoietic tissues and increase the production of haemoglobin (Figure 4) [134,136].

The main sources of excess iron deposition and iron removal by L1 in iron-loaded patients and also in patients with the anaemia of chronic disease are intracellular stored haemosiderin and ferritin polynuclear iron [147,148,149,150,151,152]. Iron removal from both haemosiderin and ferritin by L1 has also been shown in vitro, suggesting that the process is slow and that only a small portion of the total amount of iron from the proteins could be mobilised within 24 h incubations [153,154,155].

Different forms of interaction of L1 have also been reported with many other iron-containing proteins, thereby affecting their function or associated metabolic pathways. These forms of interactions may not involve iron removal from the proteins but rather other aspects of the iron-containing proteins, including the associated intracellular iron pool from which iron molecules are “in transit” and utilised for the turnover of related iron proteins [156,157]. For example, tertiary structural changes but no iron removal or oxidation of iron in haemoglobin were reported in in vitro studies with L1 [158]. In other studies, the partial inhibition of cyclooxygenase and lipoxygenase was observed through a mechanism of intracellular iron pool depletion and a reduction of protein turnover in the presence of L1 [111,159,160]. Similar mechanisms appear to be involved with other iron-containing proteins, including hydroxylases involved in a variety of functions, such as collagen synthesis, and also ribonucleotide reductase, a key enzyme for DNA synthesis and a major target for cancer therapeutics [161,162,163].

In addition to ribonucleotide reductase, several other proteins and transcription factors involved in cancer progression have been targeted by L1. These include the modulation of the hypoxia-inducible factor (HIF) related to hypoxia, the function of newly identified molecular species, such as the “six-transmembrane epithelial antigen of prostate, family member 4” (STEAP4) metalloreductase, and the metastasis suppressor N-MYC downstream-regulated gene-1 (NDRG1) [164,165,166,167,168]. Similarly, the modulation of zinc, which affects thousands of zinc-dependent transcription factors and hundreds of catalytically active zinc metalloproteins, by L1 may also be considered for cancer targeting [169,170]. Several other metabolic pathways involving iron proteins, such as aconitase modulating oxidative stress and affecting mitochondrial function, could also be targeted for anticancer activity by L1 [171,172].

## 5. The High Clinical Antioxidant Potential of Deferiprone

Free radicals, reactive oxygen species, and related by-products are continuously generated in humans and other organisms in normal physiological conditions and also in response to different stimuli, causes, and factors [26,27,28,29,30,173,174,175,176]. The formation of free radicals in biological systems is primarily dependent on iron and copper catalytic centres involved in redox reactions [26,27,28,29,30]. In conditions of redox homeostasis, the production and effects of free radicals are controlled by an innate antioxidant system involving many proteins, such as superoxide dismutase, biomolecules, such as glutathione, and dietary antioxidants, such as vitamins C and E [26,27,28,29,30,173,174,175,176]. In homeostatic redox imbalance, the excess, uncontrolled production of free radicals, which overcomes the antioxidant mechanisms, can lead to accelerating damage to biomolecules, tissues, and organs, which can be reversible or irreversible [177,178,179].

**Figure 5:**
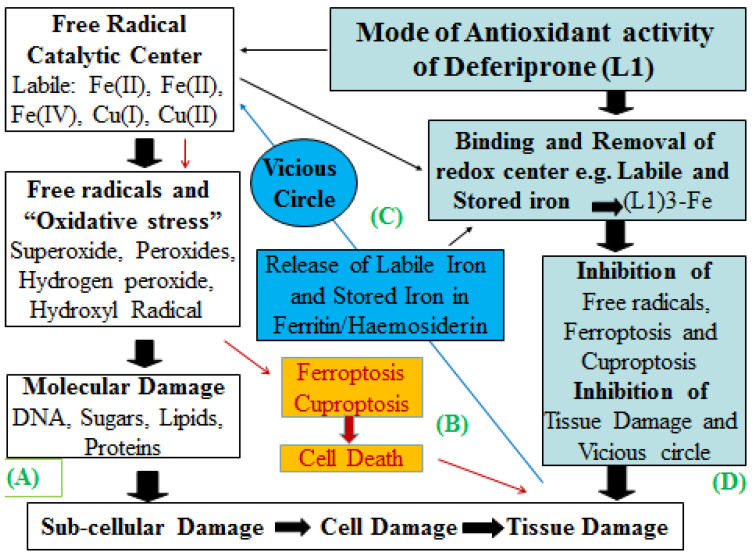
The catalytic role of iron and copper in free radical formation and oxidative stress damage, and the antioxidant activity of deferiprone. (**A**) Iron and copper catalyse the formation of free radicals, causing oxidative stress leading to molecular, subcellular, cellular, and tissue damage. (**B**) A similar pathway is followed in ferroptosis and cuproptosis where iron and copper catalyse the formation of free radicals, causing lipid peroxidation on the cell membrane and cell death. (**C**) Cell and tissue damage cause the release of iron and copper, resulting in a vicious circle of free radical production. (**D**) The antioxidant activity of deferiprone (L1) is based on the strong binding of iron and copper, which results in the inhibition of the catalytic formation of free radicals, ferroptosis, cuproptosis, and the vicious circle of tissue damage.

In particular, free radical toxicity and cascades through iron catalysis in the Fenton reaction have been shown to cause oxidative damage to all known biomolecules, including lipids, sugars, proteins, and DNA, and also tissue damage in almost all pathological conditions [27,29,177,178,179]. Within this context, iron chelating drugs could, in principle, be used as antioxidants by binding catalytic iron and inhibiting the production of free radical reactions and cascades (Figure 5) [28,157,179].

It should be noted that many different antioxidants, mostly in the form of nutraceuticals supported by a multi-billion industry, are sold daily over the pharmacy counter and other shops. Despite their popular demand and wide use, nutraceuticals are not regulated by drug authorities [179]. Similarly, despite the thousands of publications and clinical trials on the role of antioxidants in preventing oxidative stress toxicity and damage, no pharmaceutical antioxidants have yet been developed or are prescribed in medicine for the treatment of related conditions [179].

The possibility of using iron chelators as antioxidants and the potent antioxidant activity of L1 were identified at the early stages (1987) of its development when a three-screening model system was used to examine and compare the redox properties and free radical toxicity effects of different iron chelators and chelating drugs [180]. During those studies, differences in the antioxidant potential were identified among the various chelators. However, in contrast to the antioxidant effects of, for example, L1 and DF, the chelating drug ethylnediaminetriacetic acid (EDTA) has been shown under the same conditions to increase free radical toxicity and damage (Figure 1) [180]. Since then, hundreds of in vitro, in vivo, and clinical studies, as well as disease models, have shown that L1 is the most potent antioxidant drug targeting free radical toxicity, arising mainly from the Fenton reaction [179,180,181,182,183,184,185]. Similar inhibition of free radical toxicity arising from Fenton-like copper catalysis has also been shown by L1 in different in vitro experimental models of copper redox toxicity [186,187].

There have been further developments in the biological role of iron and free radicals, which have attracted the attention of a wide spectrum of biological and clinical investigators in the last 10 years following the discovery of ferroptosis, a newly identified form of programmed cell death, which is different from apoptosis and necrosis [188,189]. Among the main characteristics of ferroptosis are the induction of cell damage and death through peroxidation of cell membrane lipids caused by iron-catalysed free radical reactions and the involvement of associated metabolic pathways of iron metabolism and free radical pathology [188,189,190,191,192]. Ferroptosis has been identified in many pathological conditions, including cancer, COVID-19, and many other infectious, kidney, cardiac, and neurodegenerative diseases [193,194,195,196,197,198,199,200,201,202,203,204]. A different form of cell death with similar effects to ferroptosis is cuproptosis, where copper instead of iron is implicated in programmed cell death [205,206,207,208,209,210,211]. The inhibition by L1 of both iron- and copper-induced oxidative stress toxicity, is another unique property of the drug, which can be utilised for the design of new strategies and the development of new pharmaceuticals for the control of both ferroptosis and cuproptosis, which have been recognised in many diseases (Figure 5).

Several other unique characteristics in relation to the antioxidant pharmaceutical potential of L1, in addition to the prevention of iron and copper free radical toxicity, include the rapid drug antioxidant activity effects and its wide accessibility to most organs. In this context, L1 has been shown to inhibit the pro-oxidant effects of vitamin C, or ascorbate, a widely used nutraceutical and a key dietary molecule involved in redox reactions under normal and disease conditions (Figure 1) [212,213]. Similarly, the ability of L1 to cross the blood–brain barrier supports the possibility of unique antioxidant activities for many neurodegenerative and other brain diseases of free radical pathology and also for malignant, microbial, and other diseases [214,215,216,217].

A typical example of the clinical iron chelation–antioxidant potential of L1 is related to its widely studied beneficial effects on the hearts of iron-loaded patients, which was the major cause of mortality in thalassaemia and other transfusional iron-loaded conditions before the introduction of the drug. In extensive clinical investigations, L1 was shown to remove all excess toxic iron from the heart, resulting in the substantial improvement of cardiac function [77,83,84,85,97,98,99,100,101]. Moreover, it has been shown that the long-term use of L1 significantly enhanced left ventricular ejection fraction (LVEF) and improved the antioxidant status of the patients [86,87,88,89,218]. Similar effects have been observed in other categories of iron-loaded patients with cardiac problems in addition to thalassemia patients [219,220,221,222]. Further studies on the cellular level have suggested that the LVEF improvement is related to the antioxidant effects of L1 on endothelial cells [223,224]. Similar improvements in the antioxidant status, such as increases in glutathione levels and also in cellular function, were observed in the red blood cells of iron-loaded patients and also in animals treated with L1 [68,183,218,225,226].

Overall, it appears, in general, that L1 could prevent, delay, or reverse oxidative-stress-toxicity-related tissue damage caused by iron and copper catalytic action (Figure 5). New strategies could be developed whereby the antioxidant activity of L1 for minimising tissue damage in the heart and other organs could be further enhanced by optimising dose protocols and also by designing new antioxidant drug combinations targeting different molecules or metabolic pathways of oxidative stress toxicity. One such combination could involve L1 and N-acetylcysteine, which target Fenton-like reactions and enhance glutathione efficiency, respectively [179]. Most importantly, the potential inhibition of ferroptosis and cuproptosis by L1 reported in many diseases opens new horizons for its wide application in medicine and increases the prospects of its use as a universal antioxidant drug.

## 6. Repurposing of Deferiprone for the Treatment of Non-Iron-Loaded Diseases

The search for new pharmaceuticals for the treatment of many diseases with no current effective therapies, such as many types of cancer, Alzheimer’s disease, and Parkinson’s disease, as well as many orphan diseases, such as malaria, is a priority for the patients affected, their relatives, and society as a whole. Similarly, the length of timing for the introduction of new pharmaceuticals for such diseases is also very important, because their speedy introduction may save millions of lives each year. In this context, the repurposing of existing drugs for the treatment of such diseases may offer a much-needed solution in contrast to the development of new drugs, which usually takes much longer for the fulfilment of related regulatory requirements [12]. Some of the promising groups of approved drugs for repurposing and use in other diseases are chelating drugs and, especially, L1 [12,19,20,21,35].

The drug repurposing prospects of L1 for use in diseases other than transfusional iron overload began within a few years of the initiation of clinical trials with L1 and continue until now, involving different categories of patients with normal iron stores [227,228,229,230,231]. Most of the new categories of targeted patients are in relation to abnormalities of iron metabolism, iron and other metal toxicity, cancer, and infectious and other diseases, as originally proposed in 2003 [231]. In almost all these cases, L1 was selected for clinical studies in different non-iron-loaded categories of patients based on a risk/benefit assessment, preclinical and initial clinical results and effects in iron-loaded patients, and also as a reflection of L1′s overall safety and efficacy potential [41]. In particular, the background information on safety and efficacy was obtained from many preclinical studies involving many in vitro, cell, and animal findings, e.g., in mice, rats, rabbits, guinea pigs, and dogs, where there were some interspecies differences [67,68,69,70,71,72,73,123,232,233].

A major encouragement for initiating the repurposing of L1 in non-iron-loaded conditions was mostly in relation to the safety and efficacy of L1 observed in many iron-loaded categories of patients with various underlying conditions and different drug treatments [65]. Among the iron-loaded categories of patients treated with L1 were beta-thalassemia major, beta-thalassemia intermedia, HbE beta-thalassemia, HbS beta-thalassemia, sickle cell anaemia, myelodysplastic syndrome, aplastic anaemia, Fanconi’s anaemia, Blackfan–Diamond anaemia, pyruvate kinase deficiency, idiopathic hemochromatosis, iron overload in haemodialysis, juvenile hemochromatosis, etc. [49,50,51,52,53,54,55,56,57,58,59,62,64,110,218,219,220,221,234].

The prospects for the use of L1 in non-iron-loaded categories of patients increased substantially following the achievement of normal iron stores in iron-loaded thalassaemia patients using L1 and L1/DF combinations and their maintenance using L1 monotherapy for more than 100 patient years. This achievement was based on personalised drafted dose protocols, which signalled a new era in the complete treatment of transfusional iron overload in thalassaemia using chelation therapy [98,99,100,101]. The characterisation of the normalisation of the iron stores in ex-iron-loaded thalassaemia patients was based on diagnostic criteria and, especially, normal levels of serum ferritin and also liver and cardiac MRI T2* [235].

The same diagnostic criteria of normal serum ferritin and also liver and cardiac MRI T2* also applied in the many categories of non-iron-loaded patients with different underlying pathologies but with normal iron diagnostic indices, such as serum ferritin. However, the iron toxicity and tissue damage identified in many non-iron-loaded categories of patients concern the presence of focal iron deposits, which were detected by MRI T2* and are of major significance in many clinical conditions [236]. In particular, focal iron accumulation in the brain with increased MRI T2* signal intensity has been detected in many neurodegenerative diseases, including Alzheimer’s disease, Parkinson’s disease, Friedreich’s ataxia, and neurodegeneration with brain iron accumulation (NBIA) [237,238,239]. In the latter, at least fifteen diseases with NBIA have been characterised due to iron deposition in the globus pallidus and the substantia nigra parts of the brain [240]. In addition to the pathogenic effects of focal iron deposits, other forms of toxic iron, such as toxic labile iron forms, have also been characterised and implicated in many other diseases of free radical pathology e.g., in diabetic and non-diabetic glomerular disease, ischaemic reperfusion injury, etc. [241,242].

Significant clinical improvements have been noted in almost all the different categories of non-iron-loaded patients treated with L1, particularly when appropriate effective doses were selectively used (Table 2). For example, in six-month follow-up studies, using L1 at 20–30 mg/kg/day in Friedreich’s ataxia patients has shown a reduction of excess iron deposition in the brain with a concomitant reduction in neuropathy and ataxic gait, without apparent haematological or neurological side effects [243]. Similarly, the reduction of iron load in the basal ganglia and a trend of slowing disease progression has been shown in NBIA patients [244,245,246,247,248] and also in Parkinson’s disease patients, where the slowing down of disease progression and improved motor function were reported in some patients [249,250]. In contrast, the use of L1 at single or repeated very low doses of 15 mg/kg/day was mostly ineffective, with disappointing results [250,251]. Several clinical studies with L1 involving different categories of neurodegenerative disease patients, including Friedreich’s ataxia, Parkinson’s disease, and Alzheimer’s disease, are currently in progress.

Promising therapeutic effects have also been observed in other non-iron-loaded categories of patients treated with higher doses of L1. In particular, L1 administered at 50 mg/kg/day for 6–9 months in 53 diabetic and non-diabetic glomerular disease patients has shown a persistent drop in the mean albumin/creatinine ratio and stable renal function in diabetic patients. In contrast, a non-significant reduction in urinary protein with no significant changes in serum creatinine was observed in non-diabetic patients [252].

Much higher doses of L1 have been used in non-iron-loaded categories of HIV-1-infected asymptomatic patients and also patients with malaria. In the former case, seven patients received 33 mg/kg three times daily, and seven patients, as well as six normal volunteers, received a dose of L1 of 50 mg/kg three times daily, with 86% of the subjects tolerating the 99 mg/kg daily dose and 61% the 150 mg/kg daily dose. It was estimated that the L1 threshold at about 150 μM did not allow a viral breakthrough for up to 35 days on-drug and also at least 87 days off-drug for a viral rebound. These studies suggested that L1 is the first low-molecular-weight drug that offered the prospect of reducing the pool of cells that harbour infection-relevant HIV-1 DNA [253]. Haematological, gastrointestinal, and hepatobiliary adverse effects with primary toxicity and an increase in serum liver enzymes were reported during the trial [253]. Promising antimalarial effects have also been observed in in vitro and clinical studies using L1 at 100 mg/kg/day in a week as monotherapy and also in combination with other therapeutics [254,255].

A major section of pharmaceutical applications of L1 involves many categories of patients affected by metal intoxication in addition to iron [256,257]. These include the toxicity and modulation of essential metals, such as copper and zinc, and the toxicity of xenobiotic metals, such as aluminium, indium, gallium, uranium, americium, and plutonium, most of which are in competition with iron (Table 2) [48,111,258,259,260,261]. The prospects of orally administered clinical applications of L1 in the detoxification of many of these xenobiotic metals, which are widely and routinely used, include radiolabelled gallium in clinical diagnostic procedures, and also uranium and plutonium used in warfare ammunition and in the nuclear industry, respectively [48,111]. Similarly, there are increased prospects in relation to aluminium intoxication by L1, considering that effective aluminium mobilisation by L1 has already been shown in renal dialysis patients and aluminium-loaded animals [48,111,229,261,262].

## 7. Future Challenges and Potential New Clinical Uses of Deferiprone

Clinical experience of the use of L1 for over 30 years and its high level of safety in thousands of iron-loaded and non-iron-loaded patients increase the prospects of L1′s wider application in many other clinical conditions, whether as monotherapy or in combination therapies with other drugs. Most of the new potential clinical applications of L1 stem from or are related to its unique properties described above and mostly involve metal binding and the modulation of activity, metabolism, and toxicity.

One of the many other areas of potential clinical development and application of L1 is related to metal ion modulation, metabolism, and toxicity associated with transferrin, which is known to affect the transport of about 40 metal ions, including essential metals, e.g., Fe, Zn, Cu, and Co, diagnostic metals, e.g., In, Ga, Gd, Tc, Ru, and Sc, and other xenobiotic metals, e.g., Al, U, Pu, and Am [136,138,257]. The effects of L1 on the transport of these metal ions by transferrin in addition to iron transfer require further investigation and development in each of the specific areas of metal ion involvement, either in metabolic pathway modulation, clinical diagnosis, or metal detoxification (Figure 4).

There are many possibilities for new drug development or applications of L1 and other drugs by identifying therapeutic targets based on the modulation of key iron and other metal-containing enzymes, as well as associated metabolic pathways or biomolecules involved in many different diseases, including cancer, inflammatory and infectious diseases [134,254,255,263,264,265,266,267,268,269]. In each case, the selective target has different characteristics, and the modulation depends on minimum requirements, such as drug accessibility of the target, appropriate dose levels for achieving effective minimum inhibitory or other activity, and duration of drug action [111,159,160,161,184].

The expanding field of free radicals and many associated diseases of free radical pathology, including those associated with ferroptosis and cuproptosis, is the subject of many investigations and also a major challenge for the introduction of new pharmaceuticals, including iron chelating drugs (Figure 5) [179]. Although a number of studies have been carried out using L1 and DF in this area, the prospect of considering and testing L1 as a universal antioxidant drug against oxidative stress toxicity, ferroptosis, and cuproptosis caused by the Fenton reaction has not yet been fulfilled [181,182,183,184,185,186,187]. The oral administration and wide range of dose protocol choices could be applied for certain cases, such as anti-ageing, where doses as low as 10 mg/kg/day could be used, whereas in emergency cases of oxidative stress toxicity, such as tissue damage, doses as high as 100 mg/kg/day for up to 2 weeks initially may be considered [179].

Drug interactions, drug combinations, and synergistic and antagonistic effects are some of the issues affecting the general pharmacological, toxicological, and therapeutic activity of drugs, including chelation therapy and L1. Furthermore, the modulation of activity of drugs and also of natural dietary or other molecules with iron binding properties has a major impact on therapeutic outcomes [212,213,214,215,270]. This is shown, for example, by the introduction of the L1/DF combination in the treatment of thalassaemia, which took many years to be developed from the original concept [50,97,271,272]. In this context, several other combination options between L1, DF, and DFRA are under investigation at present [103, 105, 273, 274, 275, 276, 277 and 278].

There are several unexplored combination therapy options in the use of L1 with chelating or other drugs in different conditions, which may offer advantages over existing therapies. Examples of such L1 combinations may involve EDTA, which is widely used in general metal detoxification and also other conditions [279,280,281]; DTPA, which has been used in thalassaemia and is currently used in plutonium decontamination in the nuclear industry [282,283,284]; and the iron, copper, and aluminium phytochelator mimosine, which could be used in thalassaemia and also other conditions (Figure 1) [270,273].

The optimisation of therapies through modulation of the mode of action and the toxicity of widely used drugs with iron binding properties could also be envisaged from advantages in the use of the unique properties of L1. For example, in the case of the anticancer drug doxorubicin, where iron is thought to be implicated in its cardiotoxic effects, the chelator prodrug dexrazoxane, which is a derivative of EDTA, has been widely used in cancer patients for cardioprotection against doxorubicin and similar drug cardiotoxicity [285,286,287]. Similar effects to dexrazoxane have been shown against doxorubicin toxicity by L1 in animal studies, and L1 may be considered for replacing dexrazoxane, especially in patients experiencing severe toxicity with this drug [285,286,287,288]. The impairment of the anticancer and other therapeutic effects of the chelating drug hydroxyurea have also been suggested as a result of the presence of excess iron, especially in multitransfused patients [289,290,291]. Similar findings have also been shown in the case of tetracyclines, where iron decreased their absorption and mode of antibacterial action [292]. Modulation by L1 of the mode of action of ascorbate, a daily nutrient and widely used iron chelator nutraceutical, has also been shown [212,213,293,294]. In particular, the inhibition by L1 of the prooxidant effects of ascorbate in the presence of iron may have enhanced therapeutic and reduced toxicity implications where ascorbate is clinically used [212,213]. The selection of appropriate L1 and other chelating drug doses in relation to the depletion of essential metals is an important parameter for avoiding related toxic side effects in future chelating drug applications [295,296,297].

There are many other areas of potential clinical use of L1 where effective therapies for different conditions have not been found and initial in vitro and in vivo findings are very encouraging. In particular, the anticancer potential activity of L1 has been identified in prostate, breast, neuroblastoma, and many other cancer types, as well as the prospect of inhibition of universal mechanisms identified in cancer progression, metastasis, and drug resistance by L1 [288,298,299,300].

The unique properties of L1, its safety record, and its antioxidant, anticancer, antimicrobial, metal detoxifying, iron, and other metal metabolism modulating and other effects are very encouraging for drug repurposing. They are also important for considering and planning the design of new therapeutic strategies, including its potential use for the treatment of many new iron-loading and other non-iron-loading diseases, as well in medical diagnostics and other applications (Table 1 and Table 2) [301,302,303,304,305,306,307,308,309,310,311,312,313,314,315]. In all these cases, a risk/benefit assessment should be considered for the use of L1 in any new disease or patient case, including different factors related to the underlying disease, the present rate of morbidity and mortality, existing treatments, and the introduction of possible combination therapies [316,317,318,319,320,321,322,323,324,325,326,327,328,329,330]. The selection of the appropriate dose protocols, the duration of treatment, and monitoring methods are also very important parameters for the assessment of L1 in such new repurposing cases.

## 8. Conclusions

Deferiprone has played a major role in the transition of thalassaemia from a fatal to a chronic disease, where the rate of survival of L1-treated patients is approaching that of normal individuals. In this context, many thalassaemia patients have become grandparents, a situation unthinkable before the introduction of L1 over 30 years ago. Similarly, selected dose protocols of L1 and also combination therapies with DF have succeeded for the first time in chelation therapy history to achieve and maintain normal iron stores in chronically transfused thalassaemia patients. The unique molecular and other properties of L1, as well as its mode of antioxidant, antimicrobial, anticancer, and metal detoxifying activity and other effects, increase the prospect of its wider evaluation and use in many clinical conditions and in medicine in general. The therapeutic effects and lack of toxicity of L1 observed in so many different clinical conditions thus far encourages, in particular, its selection and potential clinical use in many conditions with no effective treatments and high rates of morbidity and mortality, including orphan drug diseases and orphan drug patients.

## Figures and Tables

**Table 1:** Properties and mode of action of the chelating drug deferiprone.

**Chemical and physicochemical properties** Physical state: white crystalline solid. Molecular weight: 139. Molecular weight of iron complex: 470. Charge of L1 and iron complex at pH 7.4: neutral. Partition coefficient (n-octanol/water) of L1: 0.19 and iron complex: 0.05 (both hydrophilic). Stability constant (Log β) of the tris L1 iron complex: 35.
**Effects on the proteins of iron transport and storage** Iron removal from diferric transferrin in iron-loaded patients: removal of about 40% of iron at L1 concentrations of greater than 0.15 mM. Iron donation to apo-transferrin by the L1 iron complex. Iron removal from and donation to lactoferrin similar to that observed in transferrin. Dose-dependent iron removal from ferritin and hemosiderin.
**Clinical characteristics** Efficacy in iron removal is related to dose. Recommended dose in transfusional iron overload: 75–100 mg/kg/day. Recommended dose in different categories of non-iron-loaded patients: minimum single dose 25 mg/kg/day and repeated doses up to a maximum 100 mg/kg/day. Decrease of iron absorption. Differential iron removal from various organs of iron-loaded patients: preferential iron removal of excess iron from the heart but also from the liver, spleen, and pancreas in iron-loaded patients. Iron redistribution in diseases of iron metabolism: deferiprone can cause iron redistribution from iron deposits and also through transferrin from the reticuloendothelial system to the erythron in the anaemia of chronic disease. Similar effect of excess iron redistribution is observed in patients with neurodegenerative diseases with excess iron in the brain treated with L1. Increased excretion of metals other than iron: increased zinc excretion in iron-loaded patients following long-term treatments. Increased aluminium excretion in aluminium-loaded renal dialysis patients. Deferiprone glucuronide metabolite: no iron binding and no increase in iron excretion. Combination chelation therapy: combination therapies of all chelating drugs are more effective in iron excretion than monotherapies. The International Committee on Chelation of L1 and DF combination protocol causes normalization of the iron stores in thalassemia patients.
**Metabolism and pharmacokinetics** Metabolite: the L1 glucuronide conjugate. T1/2 of absorption: 0.7–32 min. T max: mostly within 1 h on empty stomach. T1/2 elimination: 47–134 min at a 35–71 mg/kg dose. T1/2 elimination of the L1 iron complex: estimated within 47–134 min. T max of the L1 iron complex: within 1 h. T max of the metabolite L1-glucuronide: 1–3 h. Route of elimination of L1-glucuronide conjugate, L1, and its iron complex: urine.

Abbreviations: L1: deferiprone. T1/2: half life time. T max: timing of maximum concentration.

**Table 2:** The unique drug properties and clinical effects of deferiprone.

**The unique drug properties of deferiprone** Simple, inexpensive, one-step synthesis and wide availability worldwide. White crystalline solid; stable at room temperature for more than 15 years. Orally effective and good compliance. Rapid absorption, appearance in blood, and wide body and organ distribution. Daily use in iron-loaded patients at high doses (75–100 mg/kg) for many years with no serious toxicity. Iron removal from all organs and especially the hearts of iron-loaded patients. Ability to cross the blood–brain barrier and remove excess iron from the brain and treat malignant, neurological, and microbial diseases affecting the brain. Iron removal from transferrin in iron-loaded patients and iron donation in non-iron-loaded patients. Potent antioxidant activity through inhibition of Fe and Cu catalytic production of free radicals. Inhibition of ferroptosis and cuproptosis involved in many diseases of free radical pathology. Use in metal intoxication diseases, including those related to Fe, Cu, Al, Zn, Ga, In, U, and Pu. Drug combination therapies with DF, DFRA, EDTA, DTPA, ascorbate, mimosine, and other chelators.
**Clinical effects of deferiprone in transfusional iron-loaded thalassaemia patients ** Complete iron removal as monotherapy or combination therapy with DF from all iron storage organs. Maintenance of normal iron stores in ex-iron-loaded patients. Efficient excess iron removal from the heart and reduction of congestive cardiac failure. Improvements in the antioxidant status, including increases in glutathione levels and in cellular function. Improvement of LVEF and endothelial cell function. Decrease in the mortality rate of thalassaemia patients and transition of thalassaemia from a fatal to a chronic disease.
**Clinical effects of deferiprone in non-iron-loaded patient categories** Renal dialysis: removal of excess iron and aluminium. Rheumatoid arthritis: increase in haemoglobin and improvement of anaemia. Malaria: fast resolution of fever and coma and rapid parasitaemia clearance. HIV: antiretroviral action. Release of innate apoptotic defense of HIV-infected cells from viral blockade. Aceruloplasminemia: removal of excess cardiac iron and improvement of cardiac function. Parkinson’s disease: removal of excess iron from the brain and improvement in motor scores (30 mg/kg). Alzheimer’s disease: removal of excess iron from the brain. Friedreich’s ataxia: removal of excess iron from the brain. Reduction in neuropathy and ataxic gait. NBIA: removal of excess iron from the brain and slowing of disease progression. PKAN: removal of excess iron from the brain. Stability of the overall clinical neurological picture. Glomerulonephritis: significant reduction in urinary protein; no significant changes in serum creatinine. Diabetic nephropathy: persistent drop in mean albumin/creatinine ratio; 9-month stable renal function. Breast cancer: eradication of cancer stem cells through selective targeting of mitochondria. Prostate cancer: inhibition of prostate cancer proliferation.

Abbreviations: DF: deferoxamine. DFRA: deferasirox. L1: deferiprone. LVEF: left ventricular ejection fraction. NBIA: neurodegeneration with brain iron accumulation. PKAN: pantothenate kinase 2-associated neurodegeneration.

## Data Availability

Data sharing not applicable.

## Abbreviations

ADMET	absorption, distribution, metabolism, elimination, and toxicity
L1	deferiprone
HPLC	high performance liquid chromatography
HIF	hypoxia-inducible factor
DF	deferoxamine
DFRA	deferasirox
HIF PHD	hypoxia-inducible factor propyl hydroxylases
LVEF	left ventricular ejection fraction
MRI	magnetic resonance imaging
NBIA	neurodegeneration with brain iron accumulation
NDRG1	N-MYC downstream-regulated gene-1
PKAN	pantothenate kinase 2-associated neurodegeneration
STEAP4	six transmembrane epithelial antigen of prostate, family member 4
UPAGE	urea polyacrylamide gel electrophoresis
